# A BCR/ABL-hIL-2 DNA Vaccine Enhances the Immune Responses in BALB/c Mice

**DOI:** 10.1155/2013/136492

**Published:** 2013-06-06

**Authors:** Yanan Qin, Hongxia Tian, Guanming Wang, Chen Lin, Yangqiu Li

**Affiliations:** ^1^Department of Microbiology and Immunology, Medical College, Jinan University, Guangzhou 510632, China; ^2^Department of Otorhinolaryngology Head and Neck Surgery, The Affiliated Hospital of Qingdao University Medical College, Qingdao 266000, China; ^3^Guangdong Academy of Medical Sciences, Guangdong General Hospital, Guangzhou 510632, China; ^4^Institute of Hematology, Medical College, Jinan University, Guangzhou 510632, China; ^5^Key Laboratory for Regenerative Medicine of Ministry of Education, Jinan University, Guangzhou 510632, China

## Abstract

The use of a DNA vaccine encoding the BCR/ABL fusion gene is thought to be a promising approach for patients with chronic myeloid leukemia (CML) to eradicate minimal residual disease after treatment with chemotherapy or targeted therapy. In this study, our strategy employs genetic technology to create a DNA vaccine encoding the BCR/ABL fusion and human interleukin-2 (hIL-2) genes. The successfully constructed plasmids BCR/ABL-pIRES-hIL-2, BCR/ABL-pIRES, and pIRES-hIL-2 were delivered intramuscularly to BALB/c mice at 14-day intervals for three cycles. The transcription and expression of the BCR/ABL and hIL-2 genes were found in the injected muscle tissues. The interferon-**γ** (IFN-**γ**) serum levels were increased, and the splenic CD4^+^/CD8^+^ T cell ratio was significantly decreased in the BCR/ABL-pIRES-hIL-2-injected mice. Furthermore, specific antibodies against K562 cells could be detected by indirect immunofluorescence. These results indicate that a DNA vaccine containing BCR/ABL and hIL-2 together may elicit increased in vivo humoral and cellular immune responses in BALB/c mice.

## 1. Introduction

Chronic myeloid leukemia (CML), with an incidence of 1.5/100,000 people, represents 15% of newly diagnosed leukemia cases in adults in China. The Philadelphia chromosomal translocation t(9;22)(q34;q11) is essential to its pathogenesis and is found in approximately 95% of patients with CML. The result of this translocation is the BCR-ABL fusion protein, which is associated with the increased ABL tyrosine kinase activity that is directly associated with leukemogenesis [1].

Imatinib mesylate (IM), which is an inhibitor of BCR-ABL-coded tyrosine kinase activity, is the first drug widely used to treat CML. The prognosis of CML was markedly improved after the introduction of ABL tyrosine kinase inhibitors (i.e., IM and its derivatives). However, a number of patients with CML die due to ABL mutation-related drug resistance and blast crisis because IM does not kill leukemia stem cells (LSCs), which persist in a majority of patients and may cause disease relapse, and clinical resistance may develop predominantly due to point mutations in the ABL kinase domain [2]. Novel tyrosine kinase inhibitors have been developed to solve the mutation problem [3, 4]; however, their binding specificity and target profiles have not been readily predicted in pathological and normal cells [5, 6]. 

The success of allogeneic bone marrow transplantation, which is attributed to graft-versus-leukemia (GVL) effects targeting leukemia cells, highlights the importance of immunotherapy in myeloid leukemia [7]. Unfortunately, it is limited by the paucity of suitable donors, and it is followed by relatively high morbidity and mortality [8]. We have come to realize that immunotherapy may result in cure of the disease. Particularly, myeloid leukemia vaccines would presumably be beneficial in eradicating minimal residual disease after treatment with chemotherapy or targeted therapy [9].

Targeted immunotherapy using leukemia vaccines has been heavily investigated. It has been shown that immunoprotection against BCR-ABL-positive leukemia cells could be induced in preclinical systems after vaccination using peptide-based and dendritic cell-based vaccines and tumor cell lysates [10–12].

In patients with CML, specific immune responses against leukemia cells have also been demonstrated by peptide-based or dendritic cell-based vaccines [13–15]. Many peptide vaccine clinical trials have been performed with limited success. It has become clear that exogenous peptides alone fail to activate effective CD8^+^ T cell levels, and if induced, they tended to be transient in patients with a weakened or tolerized immune system [16].

DNA vaccines present an attractive alternative strategy to peptide vaccination [17–19]. DNA vaccines are bacterial plasmids constructed to express an encoded protein following in vivo administration and their subsequent uptake by cells. The encoded antigen is processed through endogenous and exogenous pathways, thus leading to peptide presentation via both MHC-I and MHC-II [20]. 

DNA vaccines also offer the possibility of modifying the plasmid construct to incorporate additional immunostimulatory factors to activate directly selected immune-effector pathways. Cytokine genes as genetic adjuvants are usually added, and they can bias the immune response toward a Th1 or Th2 response. Human interleukin-2 (hIL-2) is one of the immunostimulatory adjuvants used to enhance the immune response to leukemia cells. Recombinant IL-2 can increase the antileukemic activity of donor lymphocytes, and, has been reported to be a potential enhancer of donor lymphocyte infusion (DLI). IL-2 increased the response rate with improved survival in a proportion of patients who relapsed after allo- or autolymphocyte infusions [21, 22]. The hIL-2 gene has been incorporated in many DNA vaccines [23]. Our previous research has shown that DNA vaccines containing the PML-RAR*α* fusion and hIL-2 genes could increase the immunogenicity of vaccines and induce stronger, specific cellular and humoral immune responses in mice compared with the one encoding the tumor antigen alone [24]. In this paper, the BCR/ABL-pIRES-hIL-2 DNA vaccine was constructed to assess its efficacy, and test the possibility that the immune response to vaccine could be enhanced by coexpression of hIL-2. 

## 2. Materials and Methods

### 2.1. Cell Lines and Animals

The K562 cell line was obtained from the Institute of Hematology, Jinan University College of Medicine, and it was cultured in RPMI-1640 medium (Gibico-BRL, USA) supplemented with 10% fetal bovine serum (Gibico-BRL, USA) at 37°C in a 5% CO_2_ atmosphere.

Male BALB/c mice, 6 to 8 weeks of age, were purchased from the Guangdong Provincial Medical Experimental Animal Center (animal certificate no. SCXK2008-0002), and they were bred at the Experimental Animal Center of Jinan University College of Medicine under controlled conditions and received standard laboratory chow and water according to institutional guidelines. Upon delivery, the mice were allowed to acclimatize for one to two weeks before the start of the experiment. The experiments were approved by the local Animal Ethics Committee and followed international ethical standards of conduct.

### 2.2. DNA Vaccine Preparation

The 487 bp hIL-2 fragment, which contains four exons expressing the hIL-2 core structure, was amplified as previously described [24]. Amplification of the BCR/ABL fusion gene segment was performed in cDNA from K562 cells by RT-PCR. The primers used in the present study were listed in Table 1. The following is the RT-PCR program file used: 30 cycles at 95°C for 4 min, at 94°C for 1 min, at 60°C for 1 min, and at 72°C for 1 min, one cycle at 72°C for 10 min. Briefly, the BCR/ABL segment with was designed to cover the fusion point of BCR and ABL gene corresponding to p210^BCR/ABL^(b3a2), including part of exon 14 from BCR gene and part of exon 3 and exon 2 from ABL gene (GenBank accession numbers AJ131466.1). The p210^BCR/ABL^ junction region selected contained 110 amino acids (GLYGFLNVIV HSATGFKQSS KALQRPVASD FEPQGLSEAA RWNSKENLLA GPSENDPNLF VALYDFVASG DNTLSITKGE KLRVLGYNHN GEWCEAQTKN GQGWVPSNYI), with lysine of the fusion point in the middle, 20 adjacent amino acids (aa) from the C-terminal of the BCR protein fragment, and 90 adjacent aa from the N-terminal of the ABL protein fragment. 

The amplified BCR/ABL-pIRES fragment (354 bp) was inserted into the multiple cloning site (MCS) A using *Xho I* and *EcoR I* restriction sites, and the hIL-2 segment was inserted into the MCS B site of the pIRES eukaryotic expression vector (BD Biosciences Clontech, Palo Alto, CA, USA) using *Sal I* and* Not I* restriction sites. The resulting plasmid was named BCR/ABL-pIRES-hIL-2. The plasmid pIRES-hIL-2, which contained only the hIL-2 gene, and the plasmid BCR/ABL-pIRES, which contained only the BCR/ABL gene, were also prepared. 

The nucleotide sequence of the insert was verified by sequencing. Plasmids were maintained and propagated in transformed Top10 *Escherichia coli* bacteria (Pubo Biotech, Beijing, China) in the presence of ampicillin. Large-scale plasmid production was performed using an EndoFree plasmid Giga kit (Pubo Biotech, Beijing, China) according to the protocol of the manufacturer. Plasmid purification was conducted using PureLink (Invitrogen, Paisley, UK) according to the instructions of the manufacturer, and it was assessed by spectrophotometer (OD_260_/OD_280_). All samples were tested by the limulus amebocyte lysate assay (Sigma Chemical Co., St. Louis, MO, USA) to ensure that they were free of endotoxin contamination.

### 2.3. Immunization

Mice were separated into the following administration groups by random allocation. Five mice per group were used: (1) saline control, (2) pIRES, (3) BCR/ABL-pIRES, (4) BCR/ABL-pIRES-hIL-2, and (5) pIRES-hIL-2. One day before immunization, procaine was injected in the injection sites to enhance vaccine absorption. All mice were intramuscularly administered on one side of a hind leg with 200 *μ*g of purified plasmid. At the second and fourth weeks after the first immunization, all mice groups were boosted with the same plasmid dose i.m. The animals were sacrificed 14 days after the final immunization. 

### 2.4. ELISA

Cytokine production was evaluated *in vitro* in serum samples. The IFN-*γ* and IL-4 sample concentrations were determined by commercial sandwich ELISA kits (Pubo Biotech Co., Ltd., Beijing, China) following the protocol of the manufacturer. The 450 nm optical density was measured with a BIO-RAD model 450 (BIO-RAD, Hercules, CA, USA) ELISA plate reader. A standard curve was created by plotting the mean absorbance of each standard versus the IFN-*γ* and IL-4 concentrations. The results were expressed in nanograms per milliliter by reading directly from the standard curve.

### 2.5. Flow Cytometry

Lymphocyte subset analysis (CD4^+^/CD8^+^ ratio) was conducted by flow cytometry (FCM, Epics Elite ESP, Coulter) for dual-color flow cytometric analysis. Cells collected from mice spleens were immunostained with the FITC-conjugated anti-CD4 and PE-conjugated anti-CD8 antibodies (Pubo Biotech Co., Ltd., Beijing, China). Briefly, 5 *μ*L of the appropriate monoclonal antibody was incubated with 100 *μ*L of splenic cells (1 × 10^6^/mL) for 15 min in the dark before being resuspended in phosphate buffer saline (pH7.4 PBS) and before being analyzed. 

### 2.6. Indirect Immunofluorescence Assay

For detecting anti-BCR/ABL antibodies in mouse serum, the tests were performed. After spreading k562 cells (1 × 10^6^/mL) onto glass slides as target antigen, the slides were incubated with fresh serum for 30 min followed by three 10 min PBS washes. NB4 cells, a BCR-ABL-negative cell line, used as a control. Then, addition of the FITC (fluorescein isothiocyanate) fluorescent-labeled secondary antibody (Sheep anti-Mouse, Pubo Biotech, Beijing, China), the slides were incubated for 30 min, and visualized using a fluorescence microscope.

### 2.7. Histology and Immunohistochemical Analysis

Muscle samples from all injection sites were fixed in 10% neutral-buffered formalin, embedded in paraffin, sectioned at 5 *μ*m, and processed with standard deparaffinization, rehydrated, and stained with hematoxylin and eosin (HE). For immunohistochemical (IHC) analysis, muscle sample slices without staining were quenched with 3% H_2_O_2_, blocked with 10% normal rabbit sera, after probed with rabbit anti-c-ABL polyclonal antibody (Pubo Biotech Co., Ltd., Beijing, China), Detection was done using the SuperPicTure Polymer conjugate rabbit Kits (ZYMED, Carlsbad, CA, USA) and the Stable Liquid Substrate DAB System (Tiangen Biotech, Beijing, China), followed by hematoxylin counterstaining.

### 2.8. RT-PCR

BCR/ABL and hIL-2 mRNA expression analysis was performed by RT-PCR as previously described [24]. RNA was extracted from the injected muscle tissues with standard procedures using the TRIzol reagent (Invitrogen, USA). The PCR products were analyzed in a 2% agarose gel electrophoresis.

### 2.9. Statistical Analysis

Numerical data are presented as the mean ± SD. Statistical significance of differences between the study groups was analyzed using one-way analysis of variance (ANOVA). A value of *P* < 0.05 was considered statistically significant.

## 3. Results 

### 3.1. Construction of DNA Vaccines

The hIL-2 gene fragment was inserted into site B of the pIRES and the BCR/ABL-pIRES vectors to make the plasmids pIRES-hIL-2 and BCR/ABL-pIRES-hIL-2. All constructs were confirmed to be identical to published genomic sequences by sequencing. 

### 3.2. IFN-*γ* and IL-4 Secretion

**Figure 1:**
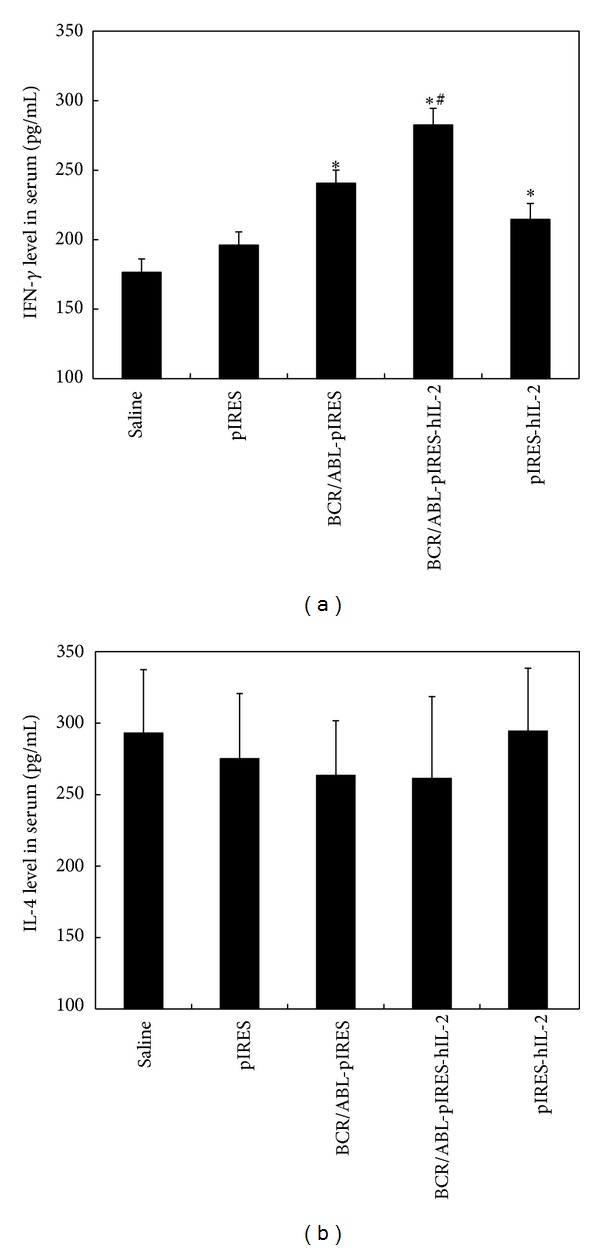
This figure demonstrates the (a) IFN-*γ* and (b) IL-4 sera secretion results. The mice (*n* = 5/group) were administered saline, an empty pIRES plasmid, BCR/ABL-pIRES, BCR/ABL-pIRES-hIL-2, and pIRES-hIL-2. The data are shown as mean ± standard deviation. *Compared with the empty plasmid group (*P* < 0.01). ^#^Compared with the BCR/ABL-pIRES group and the pIRES-hIL-2 group (*P* < 0.05). No significant difference in IL-4 secretion was found between the groups.

The mean units of IFN-*γ* and IL-4 were detected in the sera of five groups of mice immunized with different DNA constructs as shown in Figure 1. The IFN-*γ* levels of the different groups were 176.59 ± 9.53 pg/mL (saline), 196.16 ± 9.45 pg/mL (pIRES), 240.61 ± 9.54 pg/mL (BCR/ABL-pIRES), 282.55 ± 11.96 pg/mL (BCR/ABL-pIRES-hIL-2), and 214.64 ± 11.44 pg/mL (pIRES-hIL-2). The IL-4 levels of the different groups were 293.04 ± 44.36 pg/mL (saline), 275.12 ± 45.63 pg/mL (pIRES), 263.47 ± 38.19 pg/mL (BCR/ABL-pIRES), 261.27 ± 57.30 pg/mL (BCR/ABL-pIRES-hIL-2), and 294.35 ± 44.05 pg/mL (pIRES-hIL-2). The plasmids encoding BCR/ABL, hIL-2, and BCR/ABL-pIRES-hIL-2 induced a significant enhancement in IFN-*γ* secretion compared with the empty plasmid-immunized group (*P* < 0.01). The IFN-*γ* level of mice immunized with BCR/ABL-pIRES-hIL-2 was higher than that of the BCR/ABL-pIRES and pIRES-hIL-2 groups (*P* < 0.05). However, there was no significant difference in the IL-4 secretion level between the different groups (*P* = 0.682). 

### 3.3. CD4/CD8 T Cell Ratio

**Figure 2:**
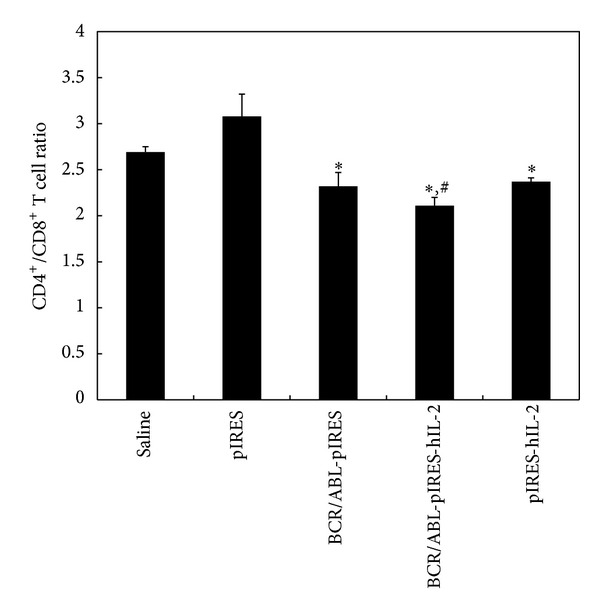
The CD4^+^/CD8^+^ T cell ratio by flow cytometry. The CD4^+^/CD8^+^ T cell ratio was lower in the BCR/ABL-pIRES-hIL-2, pIRES-hIL-2, and BCR/ABL-pIRES groups (*P* < 0.05). Additionally, the ratio of the BCR/ABL-pIRES-hIL-2 group was lower than that of the BCR/ABL-pIRES and pIRES-hIL-2 groups. *Compared with the empty plasmid group (*P* < 0.01). ^#^Compared with the BCR/ABL-pIRES group and the pIRES-hIL-2 group (*P* < 0.05).

To assess the composition of the activated T cell subsets, the cell surface markers CD4 and CD8 were detected by FCM. As shown in Figure 2, the CD4^+^/CD8^+^ T cell ratios of the different groups were 2.69 ± 0.06 (saline), 3.08 ± 0.24 (pIRES), 2.32 ± 0.15 (BCR/ABL-pIRES), 2.11 ± 0.09 (BCR/ABL-pIRES-hIL-2), and 2.37 ± 0.04 (pIRES-hIL-2). The CD4^+^/CD8^+^ T cell ratio was lower in the BCR/ABL-pIRES-hIL-2, pIRES-hIL-2, and BCR/ABL-pIRES groups (*P* < 0.05). Additionally, the ratio in mice immunized with BCR/ABL-pIRES-hIL-2 was also lower than those of the BCR/ABL-pIRES and pIRES-hIL-2 groups (*P* < 0.05).

### 3.4. BCR/ABL Antibody in Sera

**Figure 3:**
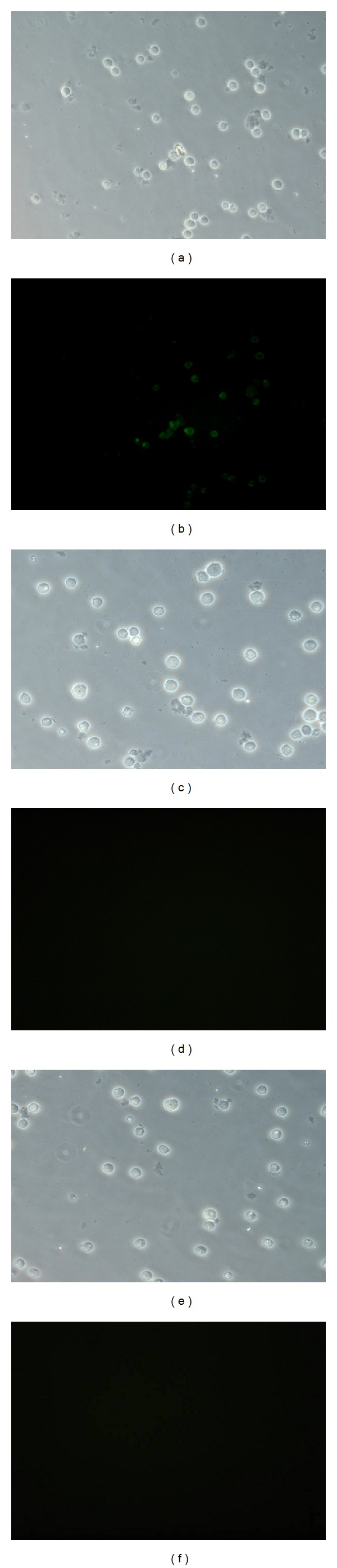
Indirect immunofluorescence of the BCR/ABL antibody in sera. K562 cells were incubated with sera from mice immunized with BCR/ABL-pIRES-hIL-2 (a) and (b), saline (c) and (d), and BCR/ABL-pIRES (e) and (f). Images (a), (c), and (e) were obtained using bright field microscopy, and images (b), (d), and (f) were obtained using fluorescence. The BCR/ABL antibody was shown in Image (b).

Specific antibodies to the BCR/ABL antigen were identified using an indirect immunofluorescence technique. Specific antibodies were only found in the BCR/ABL-pIRES-hIL-2 group (Figure 3). The sera from immunized mice were not reactive with the BCR-ABL-negative NB4 cells. The results showed that BCR/ABL antibody production could be enhanced in mice vaccinated with BCR/ABL-pIRES-hIL-2 plasmid. 

### 3.5. Transcription of DNA Vaccines In Vivo

**Figure 4:**
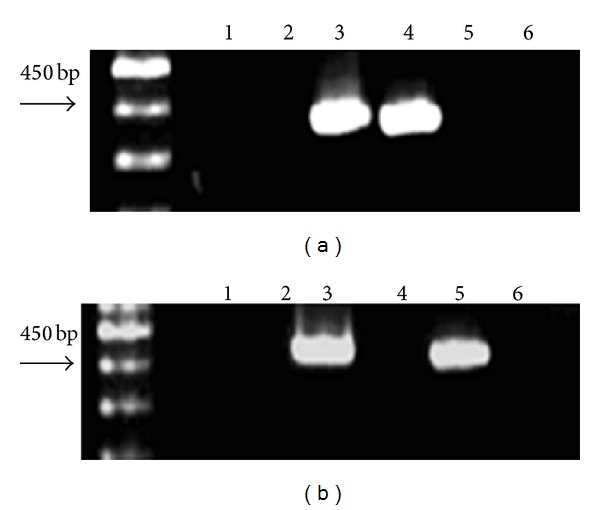
RT-PCR of muscle mRNA from the injection sites. (a) PCR products for the BCR/ABL segment (354 bp); (b) PCR products for the hIL-2 gene (487 bp). Mice were administered with saline (lane 1), the empty plasmid (lane 2), BCR/ABL-pIRES-hIL-2 (lane 3), BCR/ABL-pIRES (lane 4), and pIRES-hIL-2 (lane 5). Control for residual plasmid DNA in the injected muscle (lane 6).

We detected the mRNA expression of BCR/ABL and hIL-2 genes in mice. The BCR/ABL and hIL-2 mRNA of muscle samples from all injection sites was analyzed using RT-PCR. The results demonstrated that the BCR/ABL and hIL-2 RT-PCR products were the result of mRNA transcribed from the recombinant plasmids in muscle samples. The BCR/ABL gene was expressed in the BCR/ABL-pIRES and BCR/ABL-pIRES-hIL-2 groups (Figure 4).

### 3.6. Histological Examination of Injection Sites

**Figure 5:**
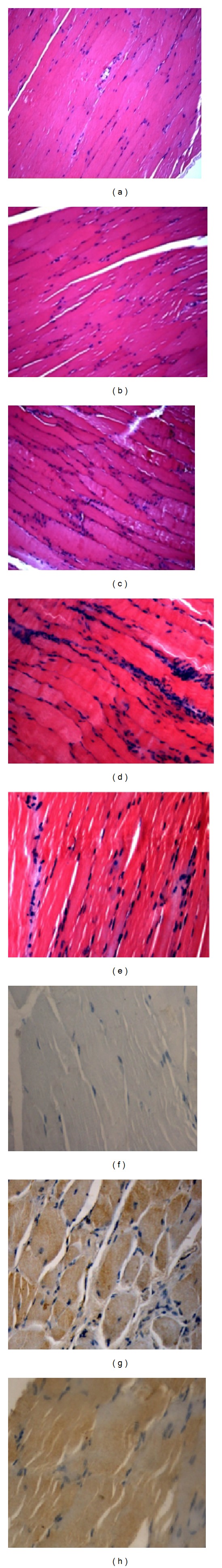
Histological examination of quadriceps muscle at injection sites. Inflammatory cell infiltration was detected by HE stain (10x). Mice were administered with saline (a), empty plasmid (b), BCR/ABL-pIRES (c), BCR/ABL-pIRES-hIL-2 (d), and pIRES-hIL-2 (e). Moderate inflammatory cell infiltration (d), mild inflammation ((c) and (e)). The expressions of BCR/ABL were detected by immunohistochemistry (DAB staining, 10x). Muscle from immunized mice with the BCR/ABL-pIRES-hIL-2 (g) and BCR/ABL-pIRES (h) showed a predominant staining pattern while the empty plasmid did not (f).

The inflammatory responses were evaluated in muscle samples from injection sites by the amount of blue (nuclear) staining. The mild-to-moderate infiltrations by inflammatory cells were observed. As shown in Figure 5, mice immunized with the BCR/ABL-pIRES-hIL-2 (d) caused moderate cell inflammatory cell infiltration, and those immunized with BCR/ABL-pIRES (c) and pIRES-hIL-2 (e) induced mild cell infiltration. Expression of BCR/ABL was additionally validated by immunohistochemistry. Muscles from immunized mice with the BCR/ABL-pIRES-hIL-2 (g) and BCR/ABL-pIRES (h) showed a predominant staining pattern while the empty plasmid control did not (f).

## 4. Discussion 

DNA-based immunization is an attractive nonviral alternative for cancer immunotherapy. The use of DNA vaccines has demonstrated the feasibility of using DNA vaccines to induce antigen-specific immune responses targeting tumor cells in preclinical and clinical trials [18, 25–27]. After vaccination, plasmid DNA is taken up by host tissue, the encoded antigen epitope binds to MHC class I molecules in the endoplasmic reticulum (ER), and it is presented on the cell surface. 

Almost all of the leukemia antigens are intracellular; thus, they are only presented as peptides in the grooves of MHC class I molecules. Only CD8^+^ T cells, which recognize these peptides, are capable of killing these leukemia cells. Thus, development of DNA vaccines that induce potent CD8^+^ T cell responses is critical. However, studies in mice have shown that the frequency of antigen-specific CTLs induced by DNA vaccines are approximately ten-fold lower compared to virally induced responses. The responses induced by DNA vaccination differ quantitatively but not qualitatively from those induced by live virus infection [28–30].

Several strategies for increasing the DNA vaccine potency have been developed in the last decade. One of these has focused on the use of various immunostimulatory molecules including cytokines or costimulatory molecules. Although numerous cytokines have been reported to have antitumor effects when administered as single-agent therapy [30–32], several studies have confirmed that the immunogenicity of DNA vaccines can be enhanced by these adjuvants [33, 34]. A panel of cytokines were added as adjuvants following DNA administration, and it was found that rhIL-2, rIL-6, rhIL-7, and rhIL-12 were able to enhance the DNA vaccination-induced therapeutic responses. For example, a plasmid expressing a tumor antigen incorporated in the hIL-2 signal peptide was more effective in protecting mice from tumor challenge [35]. Coexpression of granulocyte-macrophage colony-stimulating factor (GM-CSF) with an antigen in a DNA vaccine has been reported to result in improved immunization [36]. The rationale behind such approaches is based on facilitating T cell priming by providing additional signals through cytokine molecules.

In this study, we chose the hIL-2 gene as an immunostimulatory molecule and cloned the BCR/ABL fusion and hIL-2 genes in the pIRES eukaryotic expression vector based on our previous report [24]. Furthermore, we tested for the presence of BCR/ABL and hIL-2 mRNA by examining the injected muscles of immunized BALB/c mice by RT-PCR and the presence of BCR/ABL expression by IHC. These results show that the plasmid encoding the BCR/ABL fusion and the hIL-2 genes were successfully created. 

Cytokine production is one of the principal responses of T cell to antigen recognition. IFN-*γ* is a homodimeric protein produced by NK, CD4^+^ Th1, and CD8^+^ T cells. The functions of IFN-*γ* are important in cell-mediated immunity by CD4^+^ Th1, and CD8^+^ T cells against intracellular antigens. IL-4 is the signature cytokine of the CD4^+^ Th2 subset and functions as an inducer and effector cytokine in B cells. Therefore, we detected the serum level of IFN-*γ* and IL-4 by ELISA and the ratio of CD4^+^/CD8^+^ T cells from spleen by FCM in immunized BALB/c mice. The IFN-*γ* serum level in mice vaccinated with BCR/ABL-pIRES-hIL-2 was significantly higher than that in the BCR/ABL-pIRES or pIRES-hIL-2 groups. This finding is consistent with that of the previous report regarding the use of DNA-based immunizations [18, 24]. The IL-4 serum level was not significantly different among the groups, and the ratio of CD4^+^-to-CD8^+^ T cells in mice vaccinated with BCR/ABL-pIRES-hIL-2 was significantly decreased compared to other groups. These results suggest that the BCR/ABL-pIRES-hIL-2 design is capable of inducing a more potent cellular immune response. Neither BCR/ABL nor hIL-2 alone could induce a sufficient immune response. Taken together, it appears that coexpression of the BCR/ABL fusion and hIL-2 genes exerts a synergistic effect, which can be explained by the induced BCR/ABL-specific immune responses and the enhancement in cytokine production levels. 

The only BCR/ABL-specific antibodies in BCR/ABL-pIRES-hIL-2 group have been shown by indirect immunofluorescence, which suggests that the low level of BCR/ABL-specific antibodies might be stimulated. BCR-ABL breakpoint epitopes themselves appeared to be weak immunogenes. This is compatible with the previous findings [18, 19]. Possible reason may be related to that the DNA vaccine is processed and presented mainly through MHC class I pathway, which induces the CD8^+^ T cell response. Hrusková et al. [19] reported that in BALB/c mice VLPs carrying a long fragment covering the fusion zone failed to induce antibodies reactive with that fragment of the BCR-ABL protein. The discrepancy between our data and their data could be explained by that a portion of ABL protein fragment we selected is longer than that reported by Hrusková et al. (90 amino acids to 53 amino acids). The p210^BCR/ABL^ junction region we selected contained 110 amino acids (aa105-aa214), covering lysine 125 of b3a2 breakpoint, and 90 adjacent amino acids from the N-terminal of the ABL protein fragment. Lucansky et al. [18] reported that all of the important epitopes were located in the ABL portion of the p210^BCR/ABL^ protein. Therefore, our results support this conclusion. 

Except for CD8^+^ T cells, CD4 T cell response against b3a2 or b2a2 breakpoint epitopes was also reported in a mouse model and CML patients [15, 37]. In our present study, there was a difficulty to detecting murine cytotoxic T cells, because the recognition of antigens by CTLs in a mouse model is restricted by self class I/II MHC alleles. Further investigation is needed by using humanized NOD/SCID mouse model.

In conclusion, we successfully constructed the BCR/ABL-pIRES-hIL-2 DNA vaccine, which encodes both the BCR-ABL fusion and the human IL-2 genes, and which enhances humoral and cellular immune responses in BALB/c mice in vivo. This approach may provide new perspectives in designing cytokine-adjuvant DNA vaccines for CML clinical applications. 

## Figures and Tables

**Table 1:** Sequences of primers used in PCR.

Primers	Sequences	Restriction enzymes
BCR/ABL-forward	5′-ATT*CTCGAG*ATGGGGCTCTATGGGTTTCTG-3′	*XhoI *
BCR/ABL-reverse	5′-GCC*GAATTC*CTAGATGTAGTTGCTTGGGAC-3′	*EcoRI *
IL-2-forward	5′-GGCAC*GTCGAC*ACAATGTACAGGATGCAACTCC-3′	*Sal I *
IL-2-reverse	5′-TAT*GCGGCCGC*TCAAGTCAGTGTTGAGATGATG-3′	*Not I *

Restriction sites within primers are italicized. GenBank accession no. AJ131466 (BCR/ABL) and NM_000586 (hIL-2).
